# Structural Basis for Unusual TCR CDR3β Usage Against an Immunodominant HIV-1 Gag Protein Peptide Restricted to an HLA-B*81:01 Molecule

**DOI:** 10.3389/fimmu.2022.822210

**Published:** 2022-01-31

**Authors:** Yang Liu, Jun Lei, Dan San, Yi Yang, Chonil Paek, Zixiong Xia, Yongshun Chen, Lei Yin

**Affiliations:** ^1^ State Key Laboratory of Virology, College of Life Sciences, Wuhan University, Wuhan, China; ^2^ Department of Clinical Oncology, Renmin Hospital of Wuhan University, Wuhan, China

**Keywords:** HIV, T cell receptor, CD8^+^ T cells, HLA, antigen presentation

## Abstract

In HIV infection, some closely associated human leukocyte antigen (HLA) alleles are correlated with distinct clinical outcomes although presenting the same HIV epitopes. The mechanism that underpins this observation is still unknown, but may be due to the essential features of HLA alleles or T cell receptors (TCR). In this study, we investigate how T18A TCR, which is beneficial for a long-term control of HIV in clinic, recognizes immunodominant Gag epitope TL9 (TPQDLTML180-188) from HIV in the context of the antigen presenting molecule HLA-B*81:01. We found that T18A TCR exhibits differential recognition for TL9 restricted by HLA-B*81:01. Furthermore, *via* structural and biophysical approaches, we observed that TL9 complexes with HLA-B*81:01 undergoes no conformational change after TCR engagement. Remarkably, the CDR3β in T18A complexes does not contact with TL9 at all but with intensive contacts to HLA-B*81:01. The binding kinetic data of T18A TCR revealed that this TCR can recognize TL9 epitope and several mutant versions, which might explain the correlation of T18A TCR with better clinic outcomes despite the relative high mutation rate of HIV. Collectively, we provided a portrait of how CD8^+^ T cells engage in HIV-mediated T cell response.

## Introduction

HIV replication can be suppressed efficiently to an undetectable level by antiretroviral therapy (ART), however, because of the persistence of latent viral reservoirs, it is difficult to thoroughly eradicate the virus by ART (1–6). The activation of latent HIV infected T cells in the presence of ART has been proposed to cure HIV infection, but failed (7). And the global T cell activation could be induced by some agents, which are generally too toxic to put into clinical use (8–10). Antigen-specific T cell immunity is a fundamental ‘law’ of immunology, that is, T cell responses are highly specific and are developmentally restricted to the recognition of self-HLA (11, 12) *via* the T cell receptor (TCR). Studies have shown that the immune control of HIV infections is associated with TCR clonotypes and CD8^+^ T cell clonotypes have the greater ability to cross-react with viral epitope variants (13, 14).

CD8^+^ T cells play a vital role in the anti-viral immunity (15, 16). The activation of CD8^+^ T cells depend on the recognition of short viral peptides presented by major histocompatibility complex (MHC) class-I (17, 18). The peptides presented by MHC class I molecules act as ligands interacted with TCR to initiate a cascade of activation events, ultimately activating adaptive immune response to kill pathogenic or pathogen-infected cells (19). There is an abundance of evidence to support that CD8^+^ T cells exert potent antiviral effects in HIV control. Mathematical modeling showed that CD8^+^ T cells contribute to the reduction of plasma virus in acute infection (20). Following acute HIV-1 infection, the presence of virus-specific CD8^+^ T cells showed the rapid reduction of acute plasma viremia (21). *In vitro* study showed that CD8^+^ T cells potently inhibit HIV replication (22). Genetic study showed that HLA class I alleles contributed to HIV control (23). The previous studies showed that the immunodominant p24 Gag epitopes TW10 (TSTLQEQIGW_240–249_), KK10 (KRWIILGLNK_263–272_) and TL9 (TPQDLNTML_180–188_) could be presented by HLA-B molecules to enhance the anti-viral activity of CD8^+^ T cells (13, 24–26). And multiple HLA-B alleles can present the TL9 epitope, but the frequency and pattern of TL9 epitope mutations are distinct, and have different effects on HIV-1 replication ability (27, 28). HLA-B*81:01 presented TL9 is associated with the more efficient viral control in HIV infections (27, 29), while HLA-B*42:01 presented TL9 is less protective (30, 31). Notably, their structural studies showed that TL9 presented by HLA-B*81:01 and HLA-B*42:01 exhibits the different conformations (32). Together, these studies showed that CD8^+^ T cells play a vital role in in HIV control, cure and prevention.

In this study, we investigated the mechanism of the high-affinity CD8^+^T cell response to immunodominant HIV-1 epitope Gag-TL9 by first reporting its TCR-pHLA ternary-complex structure. An unusual opening form of Vα (the β sheet usually formed by Jβ and Vβ are not formed) was used for recognizing HLA molecule. By comparing the p-HLA structures before and after binding to the TCR, we identify the structural basis for T18A TCR recognition of HLA-B*81:01/TL9 complex and discuss the role of the unique TCR recognition in immune control of HIV.

## Materials and Methods

### Peptides

The HIV Gag p24 TL9 peptide (TPQDLNTML180-188), the escape variant Q182S, Q182T, T186S, and Q182S/T186S TL9 peptide were synthesized at > 95% purity, were synthesized at GL Biochem corporation and confirmed by high-performance liquid chromatography.

### TCR and HLA Protein Expression, Refolding and Purification

T18A TCR were bacterially expressed and refolded as previously described (33–35). For class I MHC, recombinant HLA-B*8101 and β2-microglobulin were expressed as inclusion bodies in Escherichia coli (36). HLA folding and assembly from inclusion bodies was performed according to standard procedures (37). In brief, the α- and β-chains of TCR, the heavy chain and β2m of HLA were expressed separately as inclusion bodies in a BL21 Escherichia coli strain. The inclusion bodies were washed three times and resuspended in 8M urea, then mixed into a cold refolding buffer. For TCR refolding, 1:1 ratio of α and β chains were diluted into 50 mM Tris (pH 8.3), 2 mM EDTA, 2.5 M urea, 0.5mM oxidized glutathione, and 5mM reduced glutathione. For pMHC refolding, 1:1 ratio of HLA-B*81:01 or B*42:01 heavy chain and β2m were mixed into 100mM Tris-HCL (pH 8.3), 2mM EDTA, 400mM L-arginine-HCl, 0.5mM oxidized glutathione, and 5mM reduced glutathione. Peptides were dissolved in DMSO and injected into the refolding buffer of five molar excess folds. TCR and pMHC complexes were incubated in refolding buffer for 74h and 48h at 4°C, respectively. TCR and pMHC proteins were dialyzed and further purified *via* anion exchange chromatography (HiTrap Q HP; Mono Q; GE Healthcare) and size-exclusion (Superdex 200; GE Healthcare) as described previously (38, 39). The purified protein was buffer-exchanged to 10 mM Tris-HCl, pH 8.0 and concentrated to 10 mg/ml for crystallization.

### Crystallization and Diffraction Data Collection

Protein crystals of TCR-pMHC complexes were grown at 20°C using the sitting-drop vapor diffusion technique. The T18A in complexes with HLA B*81:01 and Gag TL9 peptide was crystallized in the presence of 0.2 M Potassium chloride, 0.05 M HEPES, 35% v/v Pentaerythritol propoxylate (5/4 PO/OH), pH 7.5. For cryoprotection, protein crystals were soaked in 20% glycerol/80% mother liquor for 15s and frozen into liquid nitrogen. Data were collected at the BL19U1 beamline from Shanghai Synchrotron Radiation Facility and process with the software package HKL2000. The structures were solved by molecular replacement method using PHENIX.phaser and refined by PHENIXrefine program. Manual refinement was running in Coot. The visualization of structures was performed in PyMol and the data was deposited in the Protein Data Bank with PDB ID 7DZN.

### Surface Plasmon Resonance

The SPR assays were performed as described earlier (40–42). Briefly, the protein was buffer -exchanged into PBS and biotinylated for 1h at room temperature. The T18A TCR was fixed on the streptavidin-coated flow-cell surface of a SA sensor chip and the pMHC complexes were used as analyte. pMHC proteins was spanned by injection in concentration ranges of 0.5–250 μ M, and the equilibrium affinities were measured in 10mM HEPES, pH 7.4, 500mM NaCl, 1%BSA, and 0.02%TWEEN20 at 25°C on the Octet QKe system (ForteBio). The Kd was determined by the fitting of a single-ligand binding model.

## Results

### The Overview of Crystal Structure of T18A TCR/HLA-B*81:01/TL9 Complexes

**Figure 1:**
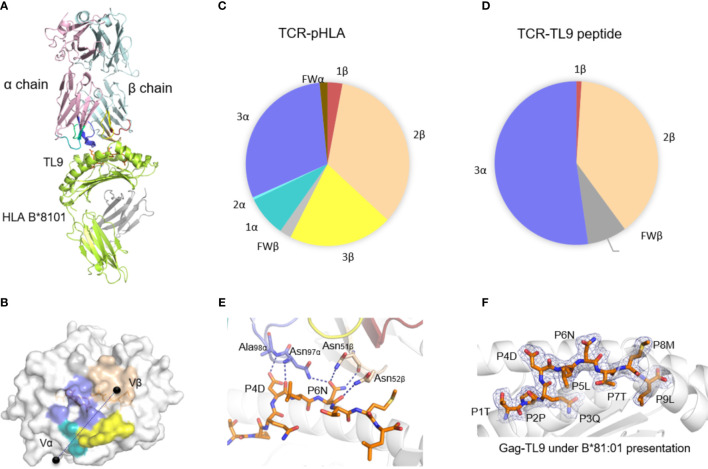
The structure of T18A TCR/HLA-B*81:01/TL9 complex. **(A)** The T18A TCR (T18Aα in pale pink, T18Aβ in pale cyan) recognize TL9 epitope presented by HLA-B*81:01. **(B)** The footprint of T18A TCR on the surface of HLA-B*81:01-TL9 complex. **(C** P Pie charts show the contribution of TCR segments toward the pHLA complex. **(D)** Interactions of TCR towards peptide. **(E)** Detailed interactions of T18A TCR with Gag-TL9 epitope in the context of HLA-B*81:01. Blue dashes denote hydrogen bonds; peptide amino acids are indicated in single-letter abbreviations and TCR residues are labeled in three-letter abbreviations. The colors correspond to TCR segment showed in pie chat. **(F)** Refined maps (2Fo-Fc) of the peptide in HLA-B complexes. The HLA molecules are represented in cartoon, and the peptides are represented as stick.

The general aspect of T18A TCR interaction with HLA-B*81:01/TL9 was shown in **Figure 1A** and the statistics of the crystal was described (**Supplementary Table 1**). The T18A TCR accommodated peptide-HLA complexes in a similar traditional diagonal manner, with a total buried surface area (BSA) (43) of 1732.6 Å^2^ in HLA-B*81:01 background which fell within the range of known BSA (44). The contact footprint of the complementarity determining region (CDR) loops at the TCR-pHLA interface was shown in **Figure 1B**. In the TCR-pHLA complex, the CDR loops contributed to the interaction were not equal, CDR2β, CDR3α and CDR3β loops were the major contributors (34%, 30% and 21% BSA) to this interaction (**Figure 1C**). Hydrogen bonds and salt bridge (CDR3β-D100 with HLA-B*81:01-R153) were observed at the interface of the complexes (**Supplementary Table 2**). TL9 peptides contributed 16% to the BSA in the HLA-B*81:01 complex. In the interaction between T18A TCR and HIV-1 Gag-TL9 epitope presented by HLA-B*81:01, CDR2β (amino acid sequence: FNNNVP) and CDR3α (amino acid sequence: VRGLNNAGNML) were the dominant contributors, which were characterized by strong hydrogen bond interactions involving multiple asparagine. Interestingly, the CDR3α and CDR2β of T18A sat above the peptide in the complex and dominated the interaction between TCR and peptide (CDR3α 52%, CDR2β 39%) (**Figure 1D**). As shown in **Figure 1E**, Asn97 and Ala98 residues of CDR3α loop formed a hydrogen bond network with the peptide-4-Asp (P4D) of TL9 peptide, while Asn97 formed a hydrogen bond with the side chain of peptide-6-Asn (P6N). The Asn51 and Asn52 of CDR2β loop formed three hydrogen bonds with the side chain and backbone of P6N. The electron density maps of the TL9 in HLA-B*81:01 presentation upon TCR binding was shown (**Figure 1F**). In general, most of the known TCRs use CDR3α and CDR3β to accommodate the various epitopes. However, CDR3β in T18A complex was functionally different from that of any other TCRs. T18A adopt a docking angle of 43° across the antigen-binding groove in the complex, and few dramatic conformational changes of the TCR on the pHLA surface was found.

### The Detailed Aspects of T18A TCR Recognition of HLA-B*81:01/TL9 Complex

**Figure 2:**
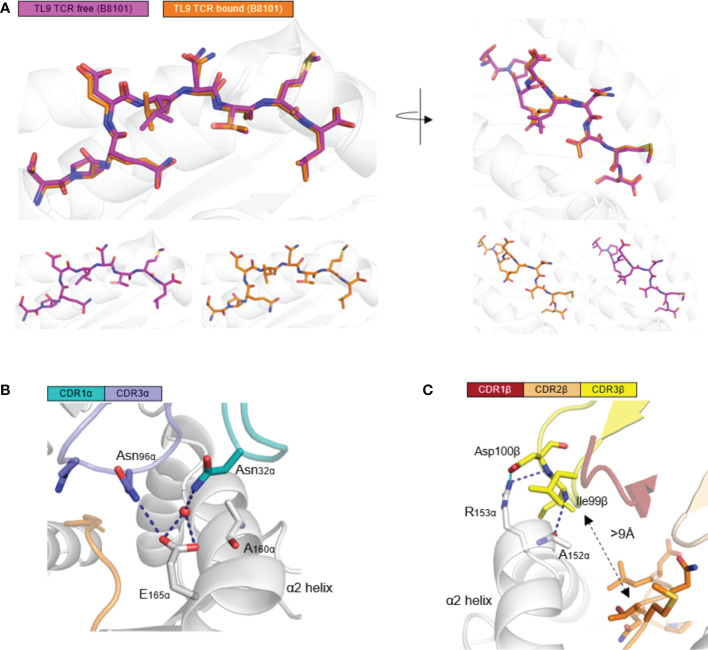
HLA-B*81:01/TL9 complex binds to T18A TCR with no conformational change. **(A)** the conformational change of HLA-B*81:01/TL9 complex after T18A TCR engagement. **(B, C)** the interactions between T18A TCR and HLA-B*81:01/TL9 complex. TPQDLNTML peptide presented by HLA-B*81:01 (PDB: 4U1I) in peptide-MHC complexes.

Next, we aimed to investigate the configuration change of TL9 peptide before and after TCR engagement. Firstly, the backbone of TL9 peptide from two complexes (HLA-B*81:01-TL9 and HLA-B*81:01-TL9-T18A) were overlapped. Secondly, the TL9 backbone of TCR free, remained the same conformation when compared to TCR bound. The side chains of the TL9 peptide were overlapped. HIV Gag-TL9 epitope exhibits the same conformation during the binding of T18A TCR (**Figure 2A**). CDR3α Loop spanned the antigen-binding cleft and contacted with peptide and HLA α2 helix. Asn96 of CDR3α and Asn32 of CDR1α interacted with E165 of HLA-B*81:01, respectively (**Figure 2B**). CDR3β loops were located above HLA α2 helix, and were far away from the peptide side chains with the distance about 9 Å. The CDR3β formed salt bridges between Asp100 and R153 of the HLA molecule, while Ile99 formed hydrogen bonds with R153 and A152 of HLA α2 helix (**Figure 2C**). CDR3β formed strong contact with HLA α2 helix, but unexpectedly not peptide.

### Unusual Role of TCR CDR3β: No Contact to the Peptide

**Figure 3:**
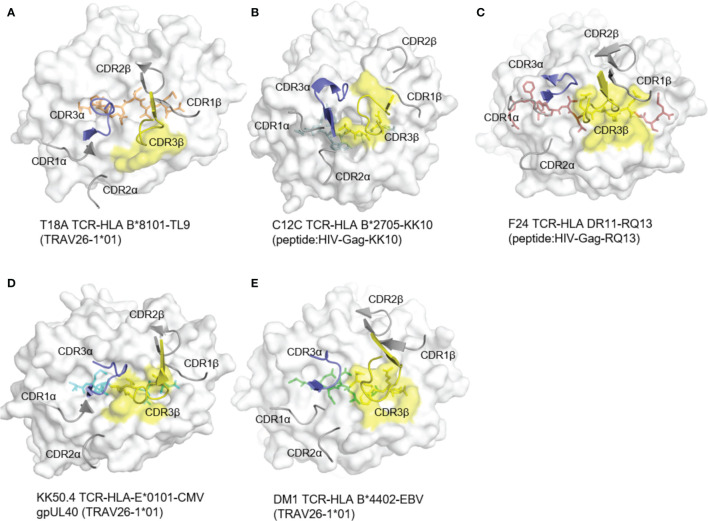
The rare docking mode of T18A CDR3β on α2 helix of the HLA but not the peptide. The foot print of TCR CDR3β on p-MHC complexes are colored in yellow from 5 different recognition profiles. **(A)** The foot print of T18A TCR CDR3β on p-HLA complex. **(B–E)** CDR3β use in other 4 structures, C12C TCR (PDB: 4G8G), F24 TCR (PDB: 6CQL), KK50.4 TCR (PDB: 2ESV) and DM1 TCR (PDB: 3DXA). Peptide in each panel is shown in stick, CDR loops are shown in cartoon, and MHCs are shown in surface view.

Generally, in T cell receptors, CDR3 regions, which contact with varied antigen peptides, are highly diversified, while CDR1 and CDR2 loops, which mainly contact with less varied HLA molecules, are less diversified. In the docking of T18A TCR toward HLA-B*81:01, however, CDR3β formed no contacts to the peptide and focused on the α2 helix of HLA (**Figure 3A**). Specifically, CDR1α interacts with HLA and CDR3α interacts with peptide and HLA. CDR3β totally interacts with HLA and does not interact with peptide. In response to the situation, parts of the CDR2β engages in the interaction with the TL9 peptide for the compensation. The complete analysis of the contacts between T18A TCR and TL9/HLA-B*81:01 complex is shown in the **Supplementary Table 3**.

To identify the role of CDR3β in other systems, we examined reported TCR-pHLA ternary structures from IEDB/3Dstructure database (38, 39, 45, 46) and PDB database (47). We checked more than 260 published mouse and human TCR structures, involving 129 different TCRs (**Supplementary Table 4**). In all of these, CDR3β interacts with peptide and MHC ligands, and most of them mainly focused on the peptide (**Figures 3B–E**). Next, we analyzed the detailed structure of CDR3β (**Supplementary Figure 2A**). In this case, CDR3β formed 2 hydrogen bonds and a salt bridge with HLA residues R153 and A152. Moreover, as CDR3β of T18A swam away from the HIV peptide, CDR2β replaced the normal role of CDR3β, CDR3α and CDR2β formed hydrogen bonds with the peptide (**Supplementary Figure 2B**). Then, we compared T18A CDR3β with those from other HIV recognition. As shown in **Supplementary Figure 3**, The bias location of T18A TCR towards HLA α2 helix was different with C12C TCR recognition. The unusual location CDR3β drives the TCR swam away from the axis of antigen-binding cleft and left the CDR2β to make moderate contacts with the C-terminal of peptide. CDR2β formed three hydrogen bonds and twelve Van der Waals interactions with the peptide. The detailed analysis of the contacts between CDR2β and peptide is shown in the **Supplementary Table 3**. Thus, the unique role of the CDR3β in the T18A TCR was not to contact the peptide but to form intensive interactions with the HLA α2 helix.

### Broken of the Traditional Jα Connection to Vα in the T18A TCR

**Figure 4:**
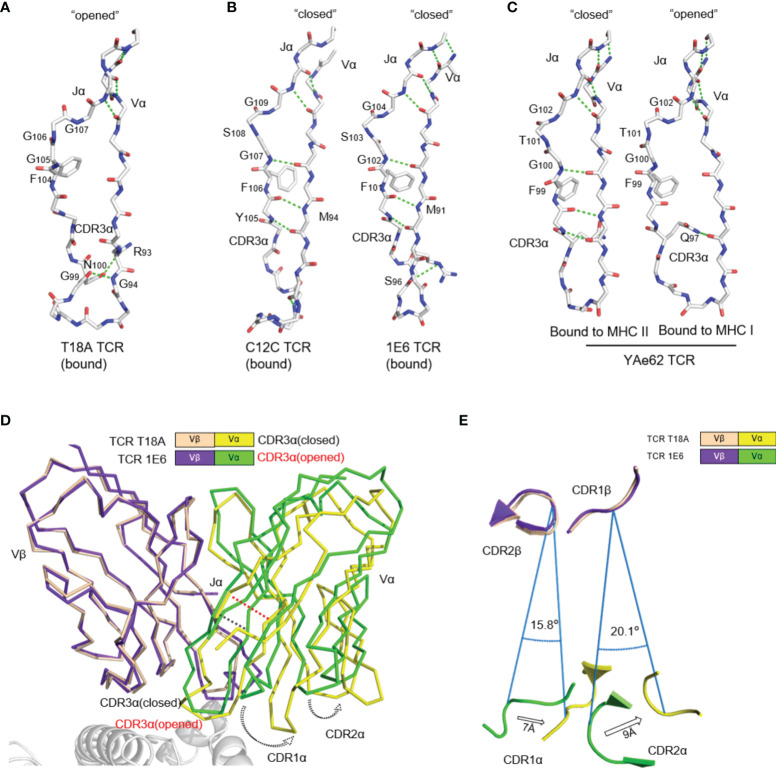
The uncommon “opened” T18A CDR3α alters the relative orientation of Vα to Vβ. **(A)** The “opened” conformation of the β sheet interactions between Vα and Jα of T18A when it is bound to B8101-pTL9. A stick representation of the protein backbone and the side chains of the FGXG conserved motif are shown. Backbone H-bonds, as well as H-bond with R93, are shown in green. **(B)** The “closed” conformation of Vα-Jα interactions of C12C TCR (PDB: 4G8G) and 1E6 TCR (PDB: 3UTS), representing traditional CDR3α conformation in most of TCR-pMHC profiles. **(C)** The disruption of Vα-Jα H bonds of YAe62 (PDB: 3C60) when it is bound to MHC II *versus* MHC I, indicating the alteration of CDR3α could expand the ability of the TCR to adapt Different MHC Ligands. **(D)** The Vα and Vβ domains of T18A and 1E6 TCR are overlaid by Vβ as similar TRBV gene is used. **(E)** A view looking down through the TCR is shown. Relative position of CDRα loops to CDRβ loops are changed due to “opened” or “closed” CDR3α. The relative distance and angle of movement is indicated.

Another interesting finding was that the traditional Jα-Vα connection was broken in T18A TCR/HLA-B*81:01/TL9 ternary structure. The core of the traditional TCR Vα domain consists of two beta-sheets, typical in V domains of the immunoglobulin family (**Figure 4B**). Unlike common “closed” Vα cores, in T18A, the disruption of the β strand made the core of Vα domain more “open” (**Figure 4A**). The lower part of the Jα-Vα interaction was destroyed, and three hydrogen bonds were broken near the conserved FGXG motif, but still preserved the interaction with the upper part of the chain. Moreover, the hydrogen bond between G99-G94 and N100-R93 fixed the lower portion of the CDR3α loop which might compensate for the broken of three hydrogen bonds. Such interruptions had been observed in mouse T cell responses, such as the “closed” conformation of the Yae62 TCR’s Vα bound to MHC I and the “open” conformation when bound to MHC II. In all of the “open” structures, the upper interaction between Jα and Vα strands was intact, but they were separated at the second glycine of the FGXG motif in a similar pattern, although different TRAV sequences were used (**Figure 4C**).

The direct consequence of this conformational change was to enlarge the distance between Jα and Vα, which finally led to the perturbation of Vα domain including CDR1 and CDR2 loops, which swang away from Vβ domain (**Figure 4D**). We superimposed T18A (TRAV26-1/TRBV12-3) and 1E6 TCR (TRAV12-3/TRBV12-4) to compare the effect of “opened” or “closed” Jα-Vα interactions on the entire TCR configuration. When Vβ domains were overlapped, the breaking of the hydrogen bond between Jα and Vα mainly affected the relative position of Vα domain to Vβ, causing Vα CDR1 and CDR2 rings to rotate by 15-20° relative to Vβ (**Figure 4E**). The opening or closing of Jα-Vα strands above the CDR3 loop altered the relative positions of Vα and Vβ CDR1 and CDR2 loops for more than 7 Å -9 Å. Additional to the traditional close conformation, this open conformation in the Vα core might enhance the recognition capacity of the TCR for versatile antigens.

### High-Affinity T18A TCR Bind to TL9 or TL9 Escape Variants Under HLA-B*81:01 Restriction

**Figure 5:**
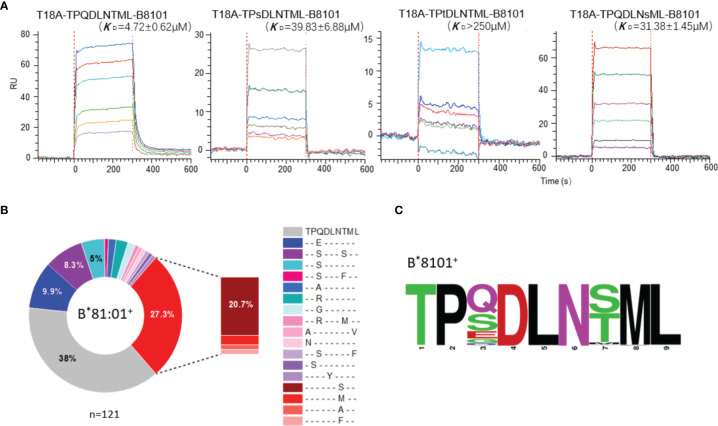
High-affinity T18A TCR bind to TL9 or TL9 escape variants under HLA-B*81:01 restriction. **(A)** SPR binding data for T18A TCR recognition of the wildtype (WT) and popular mutated TL9 presented by HLA-B*81:01. KD values range from 4.7 μM for the WT TL9 peptide to >250 μ M for the TPsDLNsML peptide. **(B)** HLA-associated variation of TL9-Gag in B8101-positve HIV infected patients. **(C)** Different escape modes in TL9 epitope are illustrated as Sequence Logo, demonstrating TL9 mutation in B8101 background is located at position 3 and 7.

Functional analysis and biophysical methods were then used to explore whether escape mutations on the Gag TL9 epitope and HLA-B*81:01 presentation affect the affinity of T18A TCR. The binding capacity of T18A TCR to different p-HLA molecules were measured by *in vitro* surface plasmon resonance (SPR). The results showed that T18A could recognize the TL9 peptide presented by HLA-B*81:01 with a high affinity (Kd≈4.7μM), and could recognize some escape variants of TL9, such as 3s-TL9 and 7s-TL9 (**Figure 5A**).

The differences in CD8^+^ T cell-mediated immunity may also influence the evolution of the TL9 epitope itself. We collected the sequencing files of >3000 HIV-1 C-clade infected patients (30, 48–51) and dissected the HLA-driven differential selection pressure (**Figure 5B** and **Supplementary Table 5**). In the context of HLA-B*81:01, the TL9 epitope mutations were mainly located at position 3 or 7 of the peptide, and the most preferred mutations were 3s-TL9 and 7s-TL9 (**Figures 5B, C**), respectively. The affinity measurement showed that mutations on these two sites of TL9 peptide could significantly reduce the affinity of TCR to pHLA molecule. Structural evidence showed that these two sites in the T18A TCR system were oriented toward the antigen-binding cleft regardless of the HLA restriction, and position 3 worked as a secondary anchored residue (**Figure 2B**). It suggested that the decreased capability of T18A TCR to the mutant epitopes may be mainly due to the decreased binding affinity of HLA molecule to TL9 variants. The occurrence of different HLA-specific adaptation patterns at TL9 epitope and significant differences in the affinity of TCRs showed the qualitatively unique CTL responses induced by closely related HLA in anti-viral immunity.

## Discussion

The MHC-restricted recognition of presented epitopes by TCRs is an essential process in the adaptive immunity against pathogens and surveillance of cancer cells. It also plays a central role in multiple immunological disorders, including allergy, autoimmune disease, and alloreactivity responses caused by organ transplantation. Although in most of the complexes TCR binds to peptide-MHC in a similar orientation, the chemical property and shapes of these interaction interface are variable and the biological response does not associate with the structural changes (minor changes might have dramatic influence on the response). The different structures representing various biological responses such as positive selection in thymus, anti-viral immune response and alloreaction still need to be reported.

The TL9 epitope was previously shown to be presented by two closely related HLA alleles B*81:01 and B*42*01 in markedly different conformations that flip several of the TCR accessible residues, and it was indicated that this difference in MHC-bound epitope conformation is responsible for the differential viral control found in B81- *versus* B42-positive patients (27, 29–32). In order to analyze why TL9 presented by HLA-B*81:01 and HLA-B*42:01 exhibited different effect in cellular immunity. We used T18A TCR to model and analyze if HLA-B*42:01 can recognize by T18A TCR. The results showed that there are some clashes in the modelling of T18A on HLA-B*42:01. Clashes on peptide involved the side chain of P4D and both backbone and side chain of P5L, which competed with Asn96 and Asn97 of CDR3α of TCR (**Supplementary Figure 1**). So, we speculated that HLA-B*42:01 may bind T18A with a weak affinity. It may be the reason of why HLA-B*42:01 is less effective in cellular immunity. However, the modeling only shows that the T18A TCR cannot bind on HLA-B*42:01/TL9 in the same way as HLA-B*81:01/TL9 did. At this moment we do not have experimental TCR-TL9-HLA-B*42:01 structure yet.

In this study, we firstly report the TCR recognition structure of HLA-B*81:01/TL9. Detail analysis and comparison revealed two interesting features of HLA-B*81:01/TL9 before and after T18A TCR engagement: 1) TL9 complexed with HLA-B*81:01 undergoes no conformational change after TCR engagement (**Figure 2A**); 2) CDR3β exhibits an interesting role that is different from that of other systems. CDR3β of T18A surprisingly focuses on recognizing the α2 helix of the HLA molecule intensively but not the peptide, which is distinct to most known TCR recognition patterns (**Figure 2D** and **Figure 3**). Subsequently the CDR2β is adopted to contact the peptide to compensate for the missing recognition of the CDR3β to the peptide, which suggested the compromised recognition for the peptide but the focused recognition for the HLA. HIV-1 sequence analysis showed that the mutation of TL9 epitope in the HLA-B*81:01 expressed individuals focused on the position 3 and 7 of the epitope (**Supplementary Table 5**). SFR assays confirmed that the T18A TCR can recognize the TL9 epitope and major position 3 or 7 mutated epitopes (**Figure 5A**). The structure of unique CDR loop patterns might explain this since T18A is more relying on HLA to supply contacts and might tolerate some different conformations of the mutated TL9 epitopes for keeping the immune surveillance. Accordingly, these findings highlight the importance of TCR structural determinants in depicting a protective clinical outcome. A molecular arm race between protective T cell response and HIV-1 mutation is suggested by these studies, the influence of host acquired immunity in genomic evolution of the HIV, therefore, might be underestimated.

## Data Availability Statement

The atomic coordinates and structure details reported in this work have been deposited in the Protein Data Bank, www.pdb.org (PDB ID codes 7DZM).

## Author Contributions

LY and YC contributed to the study design. JL wrote the original manuscript. YL conducted the protein expression, purification, and crystallization. DS did the SPR assays. JL and YL analyzed the final data and created the final figures. YL, JL, DS, YY, CP, and ZX contributed to data analysis. LY supervised the study and all authors contributed to revisions. All authors contributed to the article and approved the submitted version.

## Funding

This work was supported by the National Natural Science Foundation of China (32171210 and 31870728), Central Leading Local Science and Technology Development Special Foundation (ZYYD2020000169), and the Science Foundation of Wuhan University (2042020kfxg02, 2042016kf0169).

## Conflict of Interest

The authors declare that the research was conducted in the absence of any commercial or financial relationships that could be construed as a potential conflict of interest.

## Publisher’s Note

All claims expressed in this article are solely those of the authors and do not necessarily represent those of their affiliated organizations, or those of the publisher, the editors and the reviewers. Any product that may be evaluated in this article, or claim that may be made by its manufacturer, is not guaranteed or endorsed by the publisher.
