# Supplementary material for: Structural Basis for Unusual TCR CDR3β Usage Against an Immunodominant HIV-1 Gag Protein Peptide Restricted to an HLA-B*81:01 Molecule
Source: Front Immunol. 2022 Jan 31;13:822210. doi: 10.3389/fimmu.2022.822210 (PMC8841528; doi:10.3389/fimmu.2022.822210)
Supplement: Supplementary file 1 [file Image_1.pdf]

Supplementary Figure 1

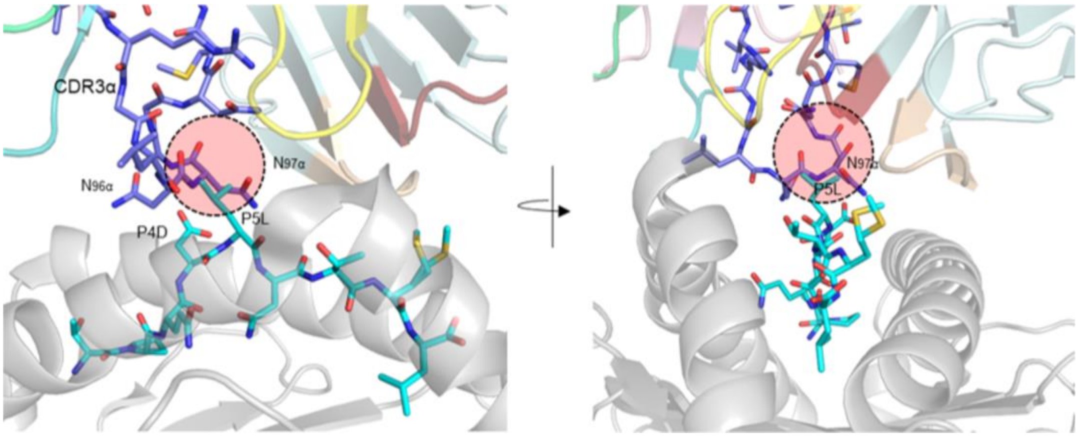

Supplementary Figure 1. Steric clashes in the modelling of T18A on HLA-B*42:01. Steric clashes between peptide (cyan) N-terminal P5L, P4D and Asn96, Asn97 of CDR3α (blue).
