# Supplementary material for: Structural Basis for Unusual TCR CDR3β Usage Against an Immunodominant HIV-1 Gag Protein Peptide Restricted to an HLA-B*81:01 Molecule
Source: Front Immunol. 2022 Jan 31;13:822210. doi: 10.3389/fimmu.2022.822210 (PMC8841528; doi:10.3389/fimmu.2022.822210)
Supplement: Supplementary file 2 [file Image_2.pdf]

## Supplementary Figure 2

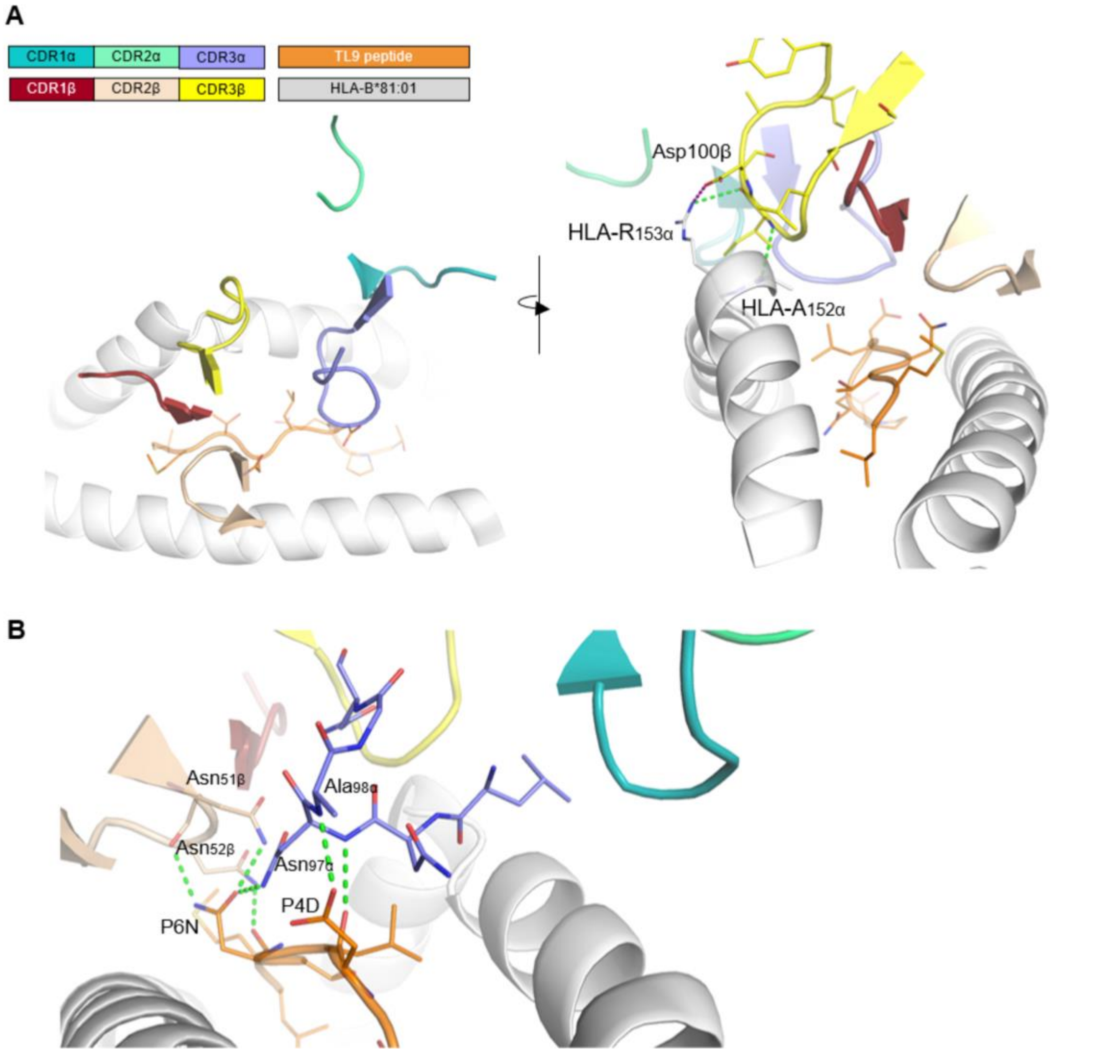


**Supplementary Figure 2. Interaction between T18A CDR loops to MHC or peptide ligands.**

**(A)** Detailed view on CDR3β loops of the T18A TCR interact with the HLA-B8101 α chain, hydrogen bonds and salt bridges are represented by green or purple dashed lines, respectively. **(B)** T18A TCR interact with the TL9 peptide via CDR2β and CDR3α loops.
