# Supplementary material for: Structural Basis for Unusual TCR CDR3β Usage Against an Immunodominant HIV-1 Gag Protein Peptide Restricted to an HLA-B*81:01 Molecule
Source: Front Immunol. 2022 Jan 31;13:822210. doi: 10.3389/fimmu.2022.822210 (PMC8841528; doi:10.3389/fimmu.2022.822210)
Supplement: Supplementary file 3 [file Image_3.pdf]

Supplementary Figure 3

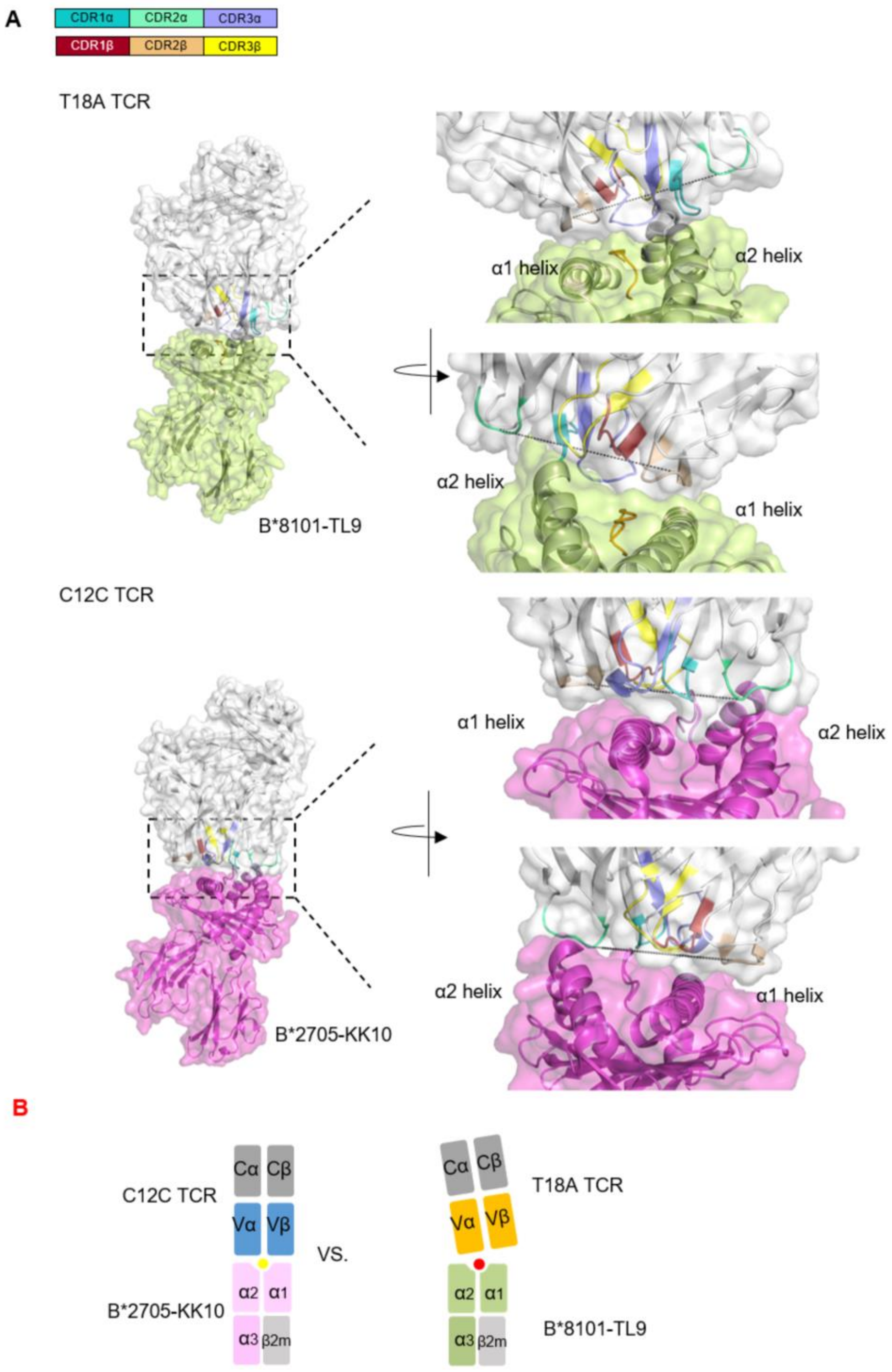

Supplementary Figure 3. Comparison of TCR docking between T18A and C12C reveals the leaning towards HLA α2 helix of T18A TCR.

(A) Top: The surface view of T18A-B*8101-TL9 recognition. T18A positions towards to the side of HLA α2 helix, and leaves CDR3α and CDR2β to interact with peptide TL9. Bottom: The surface view of C12C-B*2705-KK10 recognition. KK10 is a HIV p24 Gag derived epitope, and is immunodominant in HLAB*2705 individuals. **(B)** Cartoon representation of two TCR recognition events.
