# Supplementary material for: Structural Basis for Unusual TCR CDR3β Usage Against an Immunodominant HIV-1 Gag Protein Peptide Restricted to an HLA-B*81:01 Molecule
Source: Front Immunol. 2022 Jan 31;13:822210. doi: 10.3389/fimmu.2022.822210 (PMC8841528; doi:10.3389/fimmu.2022.822210)
Supplement: Supplementary file 4 [file Table_1.docx]

**Supplementary table 1. Data collection and refinement statistics of TCR-peptide-HLA complexes.**

|  | T18A TCR HLA-B81-Gag-TL9 |
| --- | --- |
| **Data collection** |  |
| Space group | P 43 21 2 |
| Cell dimensions a, b, c (Å) | 93.098, 93.098, 263.051 |
| Resolution (Å) | 46.55 - 2.24 |
| Total no. of observations | 113047 (11093) |
| No. of unique observations | 56526 (5547) |
| Multiplicity | 2.0 (2.0) |
| Data completeness (%) | 100.00 (100.00) |
| I/σI | 15.84 (2.00) |
| R-merge | 0.03414 (0.3732) |
| R-meas | 0.04828 (0.5277) |
| **Refinement** |  |
| Resolution (Å) | 46.55 - 2.242 (2.322 - 2.242) |
| Reflections used in refinement | 56468 (5533) |
| R-work | 0.2002 (0.2722) |
| R-free | 0.2438 (0.3701) |
| Number of non-hydrogen atoms | 7012 |
| Protein | 6664 |
| Water | 348 |
| r.m.s.d. from ideality |  |
| Bond lengths (Å) | 0.008 |
| Bond angles (°) | 0.9 |
| Ramachandran plot statistics |  |
| favored (%) | 97 |
| allowed (%) | 3 |
| outliers (%) | 0.2 |
