# Supplementary material for: Structural Basis for Unusual TCR CDR3β Usage Against an Immunodominant HIV-1 Gag Protein Peptide Restricted to an HLA-B*81:01 Molecule
Source: Front Immunol. 2022 Jan 31;13:822210. doi: 10.3389/fimmu.2022.822210 (PMC8841528; doi:10.3389/fimmu.2022.822210)
Supplement: Supplementary file 5 [file Table_2.docx]

**Supplementary table 2. Contact table of T18A/HLA-B*81:01**

| TCR segment | TCR residues | HLA-B8101 | Type of bond |
| --- | --- | --- | --- |
| CDR1α | ASN32α | GLU165α | VDW |
| CDR1α | TYR34α | GLU156α, ARG153α | VDW, HB |
| CDR3α | LEU95α | GLN157α, GLU156α, ALA160α | VDW, HB |
| CDR3α | ASN96α | GLU165α | HB |
| CDR2β | ASN52β | THR75α, LYS148α | VDW |
| CDR2β | ASN53β | GLU78α | VDW |
| CDR2β | VAL54β | GLN74α,THR75α | VDW |
| FWβ | ILE56β | ALA71α | VDW |
| CDR3β | LEU97β | ALA151α | VDW |
| CDR3β | GLY98β | ALA152α | VDW |
| CDR3β | ILE99β | AGR153α, GLN157α,GLU156α | VDW |
| CDR3β | ASP100β | ARG153α | VDW |
| CDR3β | ASP100β | ARG153α | SB |
| TCR segment | TCR residue | TL9 peptide | Type of bond |
| CDR3α | LEU95α | P5-LEU | VDW |
| CDR3α | ASN96α | P4-ASP | VDW, HB |
| CDR3α | ASN97α | P4-ASP, P5-LEU, P6-ASN | VDW, HB |
| CDR3α | ALA98α | P4-ASP | VDW |
| CDR2β | ASN51β | P6-ASN | VDW |
| CDR2β | ASN52β | P6-ASN, P8-MET | VDW |
| FWβ | ILE56β | P6-ASN | VDW |

VDW: Van der Waals interaction (cut-off at 4 Å), HB: hydrogen bond (cut-off at 3.5 Å), SB: salt bridge (cut-off at 4 Å).
