# Supplementary material for: Structural Basis for Unusual TCR CDR3β Usage Against an Immunodominant HIV-1 Gag Protein Peptide Restricted to an HLA-B*81:01 Molecule
Source: Front Immunol. 2022 Jan 31;13:822210. doi: 10.3389/fimmu.2022.822210 (PMC8841528; doi:10.3389/fimmu.2022.822210)
Supplement: Supplementary file 8 [file Table_5.docx]

**Supplementary table 5. HLA-B*08:01associated variation in TL9-Gag from studies in last decade.**

| HLA-B*81:01, N=231 | epitope | n | % |
| --- | --- | --- | --- |
|  | TPQDLNTML | 79 | 0.341991 |
|  | --X------ | 72 | 0.311688 |
|  | ------X-- | 75 | 0.324675 |
|  | others | 5 | 0.021645 |
